# A prospective, randomized, three arm, open label study comparing the safety and efficacy of PP110, a novel treatment for hemorrhoids to preparation-H® maximum strength cream in the treatment of grade 2–3 hemorrhoids

**DOI:** 10.1186/s40591-015-0043-5

**Published:** 2015-07-03

**Authors:** Ehud Klein, Ron Shapiro, Jose Ben-Dahan, Moshe Simcha, Yosef Azuri, Ada Rosen

**Affiliations:** Director of Surgery Service Line, Maccabi Health Services, Tel-Aviv, Israel; Surgery A Chaim Sheba Md ctr aff. Sackler school of medicine Tel-Aviv university, Tel-Aviv, Israel; Surgery A Meir Hospital Kfar-Saba (affiliated to Sackler Medical School, Tel-Aviv University), Tel-Aviv, Israel; Maccabi Healthcare, Tel-Aviv, Israel; Head of research Maccabi healthcare, Tel-Aviv, Israel; Department of surgery A, The E. Wolfson Medical Center, Holon and Sackler School of Medicine, The Tel-Aviv university, Tel-Aviv, Israel

**Keywords:** Hemorrhoids, PP110, Preparation-H

## Abstract

**Background:**

Hemorrhoids are a common disorder that affects the quality of life of millions of people worldwide. The effectiveness of OTC medication is limited and they mainly provide symptomatic relief. In order to treat this ailment, we formulated PP110 Gel and Wipes, as a novel treatment for hemorrhoids. PP110 is based on known active ingredients with a topical film-forming agent designed to provide physical protection and prolonged tissue contact with the active ingredients.

**Methods:**

PP110 Gel, PP110 Wipes and the comparator Preparation-H® were used on three patient cohorts. Treatment was administered once daily for PP110, and three-four times daily for Preparation-H®, for 14 days. Six different clinical parameters relating to common symptoms of hemorrhoids were monitored.

**Results:**

PP110 Gel was significantly better than Preparation-H® in reducing bleeding (Δ = 6 %), providing pain relief (Δ = 10 %) and controlling itching (Δ = 11 %). These three parameters are considered as the most common distressing symptoms for hemorrhoids patients, demonstrating that PP110 is superior to conventional treatment.

**Conclusion:**

This study demonstrated the efficacy of the PP110 Gel in treating hemorrhoids and its superiority to conventional treatments. The PP110 film-based formulation provides a slow-release mechanism and as a consequence, a prolonged therapeutic window. PP110 was both more effective in reducing hemorrhoids symptoms and more convenient to use, in that it only required application once per day.

## Background

The prevalence of hemorrhoids is extremely high in Western and other industrialized societies, with millions affected worldwide [1, 2]. In the United States it is estimated that over 10 million people suffer from hemorrhoids related symptoms [1], However, the real number of people suffering from hemorrhoids is considered much higher than that due to poor patient reporting [3, 4].

The anorectal disorder of hemorrhoids is caused by increased pressure on rectal and pelvic tissue veins, most often as a result of constipation, and is only considered a disease when symptomatic. Common symptoms include pain, itching, swelling, anal discomfort and rectal bleeding [5], all of which severely affect patient quality of life [4]. Bleeding during bowel movements, itching, and rectal pain are considered as the most common hemorrhoid symptoms [3, 6]. Hemorrhoids are most prevalent in the 45–65 year-old age group, with the same prevalence between genders. One of the ways to prevent hemorrhoids is by maintaining a healthy lifestyle based on a fiber-enriched diet with overhydrating, but this does not always help [7, 8].

Most hemorrhoids symptoms are treated with over-the-counter (OTC) preparations [2], products that typically contain active analgesics, vasoconstrictors, corticosteroids, or lubricants and provide temporary symptomatic relief. These products come in various forms including ointments, creams, gels, suppositories, and medicated wipes [9, 10]. Preparation-H® hemorrhoidal Cream (Pfizer) is arguably one of the world's best-selling hemorrhoids treatments [11].

One of the major challenges with current treatments is the demanding treatment regimen, which requires application several times a day. To overcome this problem and to improve patient compliance we devised PP110 Gel and PP110 Wipes, two novel formulations that were designed with a film-forming agent, for the treatment of grade 2–3 hemorrhoids (Grade 2: hemorrhoids which prolapse upon bearing down, but spontaneous reduction; Grade 3: hemorrhoids which prolapse upon bearing down requiring manual reduction). The film forms a protective layer, providing ‘band-aid like’ physical protection and prolonged skin contact with the active ingredients. The aim of this study is to assess the efficacy and safety of the product and to compare them to those of Preparation-H® cream in a prospective, randomized study.

## Methods

### Study design

This study was a multicenter (*n* = 7), randomized, open, 3-arm parallel group trial carried out in community clinics in Israel. Before enrolment, all participants received orally delivered and written information about the study and voluntarily signed a consent form. The trial was conducted in accordance to the ethical principles of the Declaration of Helsinki and approved by the Maccabi Healthcare Ethics Committee (Tel-Aviv, Israel; study number 2013058).

Patients were randomly assigned to the PP110 Gel, PP110 Wipes, or Preparation-H® Maximum Strength Cream treatment cohorts. It should be noted that while Preparation-H® is a common OTC product in the USA and other countries, it is unavailable for sale in Israel, thus local patients have no prior knowledge or expectation from Preparation-H®.

### Patients

Patients aged 18–70 with mildly bleeding grade 2 or 3 hemorrhoids were eligible to participate in the study. Patients with known rectal sensitivity, rectal infection, use of anti-coagulants (except Aspirin or Plavix), known inflammatory bowel disease, anal fissures, current diagnosis of cancer, and/or pregnancy were excluded from the study.

Patients in the two test arms were instructed to apply PP110 once daily for 14 days, 1–5 min prior to the first bowel movement, in order to allow the film to form and serve as a protective layer. Patients in the comparator arm were instructed to use Preparation-H® per label, i.e., 3–4 times per day, after bowel movement. Patients were contacted by phone or by means of a text message every 2–3 days, to maximize compliance and adherence.

Bleeding (the primary endpoint), pain, itching, swelling, discharge and discomfort were recorded by the recruiting physician at baseline. Pain was measured on a Visual Analogue Scale (VAS) from 0 (“No Pain”) to 10 (“Maximal Pain”), while the remaining parameters were measured on a 4-point Likert scale consisting of: 1 (“None”), 2 (“Minimal”), 3 (“Moderate”) and 4 (“Significant”). At the end of each day during the 14-day treatment period, patients completed a short questionnaire regarding their clinical symptoms. After 14 days of treatment, patients visited the clinic for a follow up visit, where the physician performed a final evaluation of the six parameters. During the same visit, patients were asked to complete a feedback questionnaire addressing treatment efficacy and overall satisfaction. A week later, they were contacted again by phone to verify their overall well-being. Safety parameters were recorded throughout the treatment and follow up periods.

### Withdrawals

Nine patients withdrew early and their data were lost. Some of them did not provide a reason (5 cases), others claimed they had lost their diary (2 cases), one claimed he did not in fact have hemorrhoids and one (from the PP110 Gel group) decided to stop using treatment since his bleeding worsened. Distribution of patients among arms (3 for PP110 Gel, 1 for PP110 Wipes and 5 for Preparation-H®) does not show significant differences. In addition, four patients were removed from the efficacy analysis due to major protocol violations.

### Materials

PP110 (Peritech Pharma LTD, Israel) is a gel or liquid based product, comprising an oil-in-water emulsion. Active ingredients (Pramoxine HCl 0.25 % and Phenylephrine HCl 1 %) are dissolved in the aqueous phase. To manufacture the Gel formulation we added a gelling agent, which is stable at high ionic strength. The oily phase mainly contains volatile ingredients with a film-forming polymer. Upon application, the volatile materials evaporate leaving the polymer attached to the tissue, along with the active ingredients. The Liquid formulation is applied to folded fabric wipes, which are then individually sealed in laminated sachettes.

Preparation-H® Maximum strength is commercially available in the USA in cream form and contains the same active ingredients at the same concentrations (Pramoxine HCl 0.25 % and Phenylephrine HCl 1 %) [12].

### Statistical analysis

Statistical analysis was performed using Analysis Of Variance (ANOVA), for each of the test arms versus the control arm (Preparation-H® treatment). Two-sided 95 % confidence intervals, as well as *P*-values were used, as estimated by the ANOVA model. All collected data was either plotted as the change from baseline (PCB) relative to the day of treatment, or calculated as the Area under the curve (AUC) for each graph.

Responders were defined as those who showed an improvement in any clinical measure between enrollment and last visit, the two recordings performed by the recruiting physicians only.

## Results

No significant inter-arm differences in baseline demographics and characteristics were observed, except for history of hemorrhoids where both test arms had a longer history than the control arm (Table 1).

**Table 1:** Patient baseline demographics and characteristics

	PP110 Gel	PP110 Wipes	Preparation-H®	All arms	*P*-Value^*^
	(*N* = 34)	(*N* = 33)	(*N* = 34)	(*N* = 101)	
Gender, n (%)					
Female	17 (50 %)	22 (67 %)	21 (62 %)	60 (59 %)	0.36
Male	17 (50 %)	11 (33 %)	13 (38 %)	41 (41 %)	
Age (Mean ± SD)	54.9 ± 11.1	53.3 ± 14.2	55.3 ± 14.6	54.5 ± 13.2	0.81
Hemorrhoid grade, n (%)					
2	17 (50 %)	14 (42 %)	23 (68 %)	54 (53 %)	0.10
3	17 (50 %)	19 (58 %)	11 (32 %)	47 (47 %)	
History of hemorrhoids, n (%)					
Less than 1 year	6 (18 %)	1 (3 %)	11 (32 %)	18 (18 %)	0.01
1 to 2 years	4 (12 %)	1 (3 %)	4 (12 %)	9 (9 %)	
2 to 5 years	4 (12 %)	12 (36 %)	5 (15 %)	21 (21 %)	
5 to 10 years	3 (9 %)	5 (15 %)	6 (18 %)	14 (14 %)	
Over 10 years	17 (50 %)	14 (42 %)	8 (24 %)	39 (39 %)	

**Fig. 1:**
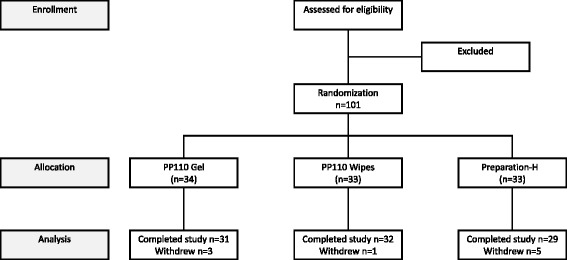
Flowchart of study inclusion, randomization, and patient disposition

A total of 101 patients were randomly assigned to one of the three treatment cohorts. Figure 1 summarizes patient enrolment, randomization, and disposition.

**Fig. 2:**
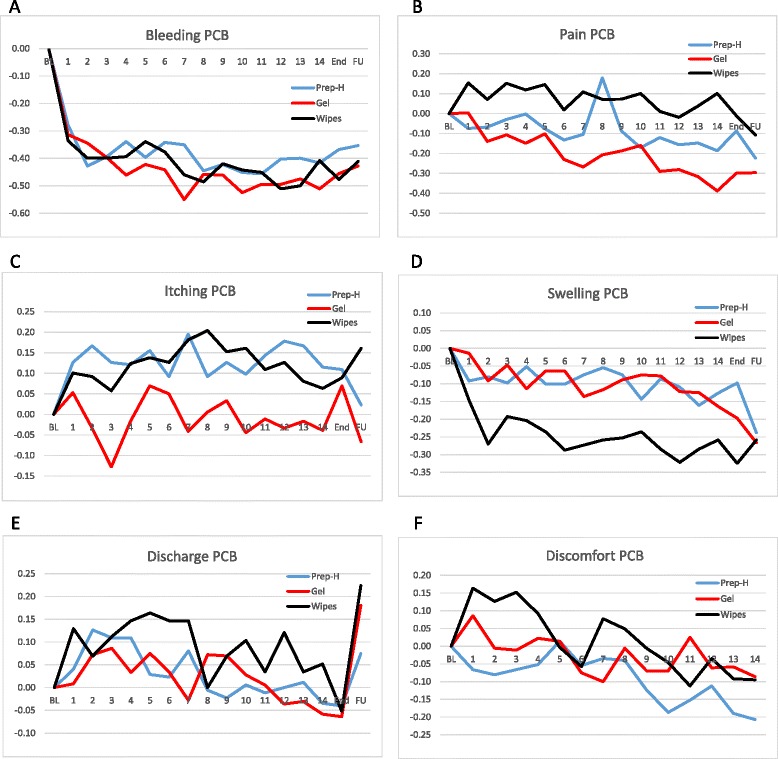
Percent Change from Baseline (PCB) in each of the six clinical parameters monitored: Bleeding (**a**), Pain (**b**), Itching (**c**), Swelling (**d**), Discharge (**e**) and Discomfort (**f**). Baseline is always defined as 0 for each arm. Days 1–14 are self-reported, End visit is evaluated by the physician, and Follow Up (FU) data is collected over the phone. PP110 Gel is always shown in red, PP110 Wipes in black and Preparation-H® in blue

When assessing the impact of the treatments on individual clinical symptoms, each treatment demonstrated a marked effect on one or more clinical parameter, which was either equal to or superior to the other tested treatments. Bleeding, which was the primary endpoint of this trial improved by more than 30 % within 1–2 days of treatment in all three arms (Fig. 2a), but was most notably affected by the PP110 Gel, which yielded a mean 45 % AUC across the treatment period. The PP110 Wipes and Preparation-H® yielded a mean 42 % and 39 % bleeding reduction AUC, respectively. These results resulted with a p-value of 0.005 for the difference between PP110 Gel and Preparation-H®, and a non-significant *p*-value of 0.2 for the difference between PP110 Wipes and Preparation-H®. PP110 Gel also provided the greatest pain relief, when compared to the other treatments, with a mean 21 % improvement, while Preparation-H® treatment led to a mean 9 % improvement and PP110 Wipes to a 6 % improvement in pain scores relative to baseline (Fig. 2b).

When applied in wipe form, PP110 treatment provided significant swelling relief, with a 24.3 % improvement relative to PP110 Gel (9.2 % improvement) and Preparation-H® (11 % improvement, Fig. 2d). For itching, no relief was shown following treatment with either PP110 Wipes or Preparation-H®, both resulting in symptom exacerbation of about 12 %; PP110 Gel on the other hand yielded an improvement of 1 % (Fig. 2c). None of the products effectively addressed discharge, with PP110 Gel and Preparation-H® showing a deterioration of 2 %, while treatment with PP110 Wipes resulted in 9 % deterioration (Fig. 2e). Finally, discomfort levels improved by 10 % following Preparation-H® treatment, while PP110 Gel treatments improved by only 2 % and PP110 Wipes was associated with exacerbation of the symptom by 3 % (Fig. 2e). In summary, PP110 Gel was significantly superior to Preparation-H® in relieving bleeding, pain and itching, while PP110 Wipes were significantly superior to Preparation-H® in addressing swelling (Table 2). However, PP110 Wipes were significantly inferior to Preparation-H® in treatment of pain and discomfort.

**Table 2:** Statistical differences in AUC between each of the test arms and the active control arm. PP110 Gel is more effective than Preparation-H® with respect to bleeding, pain and itching (in a manner that is statistically significant). PP110 Wipes are more effective than Preparation-H® with respect to swelling, but less effective with respect to pain and discomfort

Symptom	PP110 Gel vs. Preparation-H®				PP110 Wipes vs. Preparation-H®			
	Difference	Lower CI	Upper CI	*P*-value	Difference	Lower CI	Upper CI	*P*-value
Bleeding	−0.06	−0.10	−0.02	0.0059	−0.03	−0.08	0.02	0.2034
Pain	−0.12	−0.19	−0.04	0.0025	0.15	0.05	0.25	0.0023
Itching	−0.13	−0.21	−0.05	0.0008	−0.01	−0.10	0.07	0.7673
Swelling	−0.00	−0.05	0.05	0.9367	−0.15	−0.20	−0.10	<.0001
Discharge	−0.01	−0.06	0.04	0.6908	0.05	−0.02	0.12	0.1344
Discomfort	0.06	−0.01	0.14	0.0958	0.10	0.03	0.18	0.0084

When using responder analysis the results were quantitatively different but qualitatively similar. The PP110 Wipes cohort had 89.7 % bleeding responders, compared to 76.7 % in the PP110 Gel and 72.4 % in the Preparation-H® cohorts (*p* = 0.09). In pain, PP110 Gel treatment led to a 70 % response rate, compared to 44.8 % in both PP110-Wipes and Preparation-H® cohorts (*p* = 0.06). Similarly, itching responders were 30 % in PP110 Gel, and 17.2 % in both PP110 Wipes and Preparation-H®.

Satisfaction Measures: Patient comfort levels were slightly higher in the Preparation-H® cohort when compared to the PP110 Gel and Wipe cohorts (Table 3). Overall satisfaction and likelihood of future use of both PP110 Wipes and PP110 Gel were higher than Preparation-H®. Specifically, the first quartile for Preparation-H® was 2.5, whereas for PP110 Wipes it was 3 and for PP110 Gel it was 4. In both test groups, 75 % of the subjects scored likelihood of future use ≥4, whereas in the control group, 50 % of subjects provided scores below 4, half of which were below 2.

**Table 3:** Patient comfort, satisfaction and prospects of future use

Parameter	Group	Min.	1st Quartile	Median	3rd Quartile	Max.	N
Comfort	Gel	1	4.0	5.0	5.0	5	30
	Prep-H	2	5.0	5.0	5.0	5	28
	Wipes	1	3.0	5.0	5.0	5	29
Satisfaction	Gel	1	4.0	4.0	5.0	5	30
	Prep-H	1	2.5	4.0	5.0	5	28
	Wipes	1	3.0	5.0	5.0	5	29
Future use	Gel	1	4.0	5.0	5.0	5	30
	Prep-H	1	2.0	4.0	5.0	5	28
	Wipes	1	4.0	5.0	5.0	5	29

Safety: Patient monitoring included recording of treatment related adverse effects (AEs). These included Rash, burning, discharge and a non-treatment related symptom: Diarrhea (Table 4). No significant treatment related adverse effects were reported. No other safety related issues were reported.

**Table 4:** Adverse events per cohort

	PP110 Gel (*n* = 34)		PP110 Wipes (*n* = 33)		Preparation-H® (*n* = 34)	
	No.	%	No.	%	No.	%
Adverse Events - Treatment Related						
Itching	4	11.7 %	2	6.1 %	5	14.7 %
Rash	1	2.9 %				
Burning			1	3.0 %		
Discharge			1	3.0 %		
Non-Treatment Related						
Diarrhea					1	2.9 %
Total	5	14.7 %	4	12.1 %	6	17.6 %

## Discussion

Topical OTC hemorrhoid treatments have been widely used for several decades. Most of these treatments are aimed at providing symptomatic relief rather than truly altering the underlying pathophysiology of the disease. Even though these treatments are commonly used, data regarding their efficacy is sparse [13].

Preparation-H® (Pfizer) is arguably the most popular OTC antihemorrhoidal preparation in the US, and is available in many forms, including ointment, cream, gel, suppositories, and medicated wipes. It is said to provide temporary relief of acute symptoms of hemorrhoids, such as pain and itching on defecation [1, 9]. However, clinical trials in this field are scarce and no randomized controlled trials supporting its acclaimed efficacy have been documented [3]. To the best of our knowledge, this is the first publication testing the efficacy of Preparation-H® in a controlled clinical trial, or in fact in any trial.

This clinical trial evaluated the effect of PP110 Gel and medicated Wipes, designed to form a thin protective layer at the treatment site. This protective layer acts as both a physical barrier preventing irritation to the inflamed tissue and as a slow release reservoir of the active ingredients, with improved tissue contact. This supports a once-daily application regimen, which is preferred over up to 4 daily applications required for Preparation-H®.

The PP110 Gel formulation induced a statistically significant improvement, in comparison to Preparation-H®, in bleeding, pain and itching, the most common disturbing symptoms of hemorrhoids. The PP110 Wipe formulation was superior to Preparation-H® in treating swelling, although Preparation-H® was superior to PP110 Wipes in alleviating pain and discomfort. Discomfort is loosely defined and may have been interpreted differently by different patients. A clearer definition of discomfort symptoms may be advantageous in future studies.

Despite almost identical formulations, the PP110 Gel and Wipes formulations, led to significantly different clinical outcomes, which arose from the advantage provided by the protective film layer formed following application of the product. When using the Wipe formulation, the concentration of active ingredients reaching the affected area may be too low to achieve any beneficial effects. As Wipes were clearly superior over other arms with respect to swelling, this could be an interesting subject for future studies.

The lack of clinical data regarding the efficacy of OTC hemorrhoid medications has led many physicians to doubt whether they provide any significant benefit for patients [13, 14]. The current study is able to confirm their clinical effects and uncovers significant differences between treatment options for the first time.

## Conclusion

This study demonstrated the efficacy of the PP110 Gel in treating hemorrhoids. The advanced therapeutic film-based technology provides for a slow-release depot, which allows for an extended and effective therapeutic effect, together with dermal protection, and bears significant advantages over current treatments. Overall, these features may advance PP110 Gel to the forefront of next-generation anti hemorrhoidal therapies.
